# Quick creation and mapping of EMS‐induced maize kernel mutants identifies classical gene *ZmBT1* and novel gene *ZmTOP6A*

**DOI:** 10.1002/tpg2.70210

**Published:** 2026-02-23

**Authors:** Haixiao Dong, Hao Chen, Yuan Jiang, Jingzhe Zhang, Chaoyue Wang, Zhili Sun, Shengzhong Su, Shipeng Li, Hongkui Liu, Xiaohui Shan, Yaping Yuan

**Affiliations:** ^1^ Jilin Engineering Research Center for Crop Biotechnology Breeding, College of Plant Science Jilin University Changchun China

## Abstract

Maize (*Zea mays* L.) kernel mutants are valuable tools for investigating kernel development. In this study, we employed ethyl methanesulfonate (EMS) mutagenesis of pollen on five inbred lines, which displayed varying performance after treatment. Over 400 independent kernel mutants were generated, showing a wide range of defects in both type and severity. Bulked segregant analysis (BSA) combined with whole‐genome sequencing was employed to map two representative mutants. For a shrunken kernel mutant, a missense mutation (P155L) was identified in the classical *ZmBT1* gene, which encodes an ADP‐glucose transporter. For a small kernel mutant, a start‐lost mutation (M1?) was discovered in the *ZmTOP6A* gene, which encodes the DNA topoisomerase VI subunit A. Allelic verification of *ZmBT1* and *ZmTOP6A* confirmed their association with the mutant phenotypes. Furthermore, we analyzed the protein conservation, expression patterns, and subcellular localization of both genes. Our study highlights the effectiveness of combining EMS mutagenesis with BSA for mining maize kernel genes. The mutants and the identified genes will advance our understanding of maize kernel development.

## Core Ideas

- Ethyl methanesulfonate mutagenesis of pollen was performed on five maize inbred lines, resulting in over 400 kernel mutants.
- ZmBT1 was identified as responsible for a shrunken kernel mutant, while ZmTOP6A was responsible for a small kernel mutant.
- The protein conservations, expression patterns, and subcellular localizations of ZmBT1 and ZmTOP6A were analyzed.

## Plain Language Summary

Maize (corn) is an important crop around the world, used for animal feed, industrial raw materials, human food, and scientific research. Understanding how maize kernels grow is crucial for improving crop yield and making them more resilient. This study used a chemical called ethyl methanesulfonate to cause mutations and paired this with advanced gene‐mapping methods to identify the genes responsible. Over 400 maize kernel mutants were created from five maize inbred lines. Two important genes were discovered: *ZmBT1*, which controls starch production and causes shrunken kernels when mutated, and *ZmTOP6A*, a new gene that helps maintain DNA topological structure. Mutations in *ZmTOP6A* led to small kernels that could not germinate. This study offers a useful approach for finding important genes in maize, and the mutants and genes discovered will help improve our understanding of kernel growth, which could be useful for future maize improvements.

**Abbreviations**
BSA: bulked segregant analysis
EGFP: enhanced green fluorescent protein
EMS: ethyl methanesulfonate
MP: mutant pool
PCA: principal component analysis
PCR: polymerase chain reaction
SNP: single‐nucleotide polymorphism
WGS: whole‐genome sequencing
WP: wild‐type pool

## 1 INTRODUCTION

Maize (*Zea mays* L.) is the leading cereal crop worldwide and a key model organism for both forward and reverse genetics, particularly in the study of kernel (seed) development. Maize kernels are ideal for phenotypic observation owing to their manageable size, and a single ear produces several hundred kernels, enabling large‐scale genetic investigations. While transposon systems such as *Mutator* (Robertson, 1978) have been widely used to generate genome‐wide mutations in maize (Liang et al., 2019; Marcon et al., 2020; McCarty et al., 2005; Settles et al., 2007). Ethyl methanesulfonate (EMS) mutagenesis offers several complementary advantages: (1) as an alkylating agent, EMS predominantly induces C:T > G:A point mutations; (2) mutations are distributed at high density across the genome; (3) it can generate allelic series ranging from hypomorphic to gain‐of‐function mutations; and (4) it can be applied to any maize inbred line without significant limitations (Maple & Møller, 2007; Sega, 1984).

Previous studies have successfully created thousands of EMS‐induced maize mutants (Chong et al., 2025; Lu et al., 2018; Neuffer & Sheridan, 1980; Nie et al., 2021; Tran et al., 2020). Most studies have focused on treating pollen with EMS rather than seeds, due to higher mutagenesis efficiency. First, maize produces a large quantity of pollen, which is easily collected, treated, and used for pollination to generate a substantial number of seeds. Second, pollen is a single cell with a single set of chromosomes, making it more sensitive for introducing mutations compared to seeds, which are multicellular and contain two sets of chromosomes. Additionally, as a haploid stage, mutations in pollen are more easily transmitted to the next generation, while treated seeds are typically chimeric and exhibit aberrant growth.

Identification of causative mutations has also advanced considerably. Traditional positional cloning is labor‐intensive, requiring large populations and discrete markers development (Gallavotti & Whipple, 2015). With the advent of reference genomes and advancements in sequencing technologies, bulked segregant analysis (BSA) has been combined with high‐throughput sequencing methods to accelerate the identification of causative mutations (Schneeberger, 2014). For example, the SHOREmap pipeline was developed for *Arabidopsis* (*Arabidopsis thaliana* (L.) Heynh.) (Schneeberger et al., 2009). The MutMap pipeline was developed for rice (*Oryza sativa* L.) (Abe et al., 2012), which has evolved into MutMap+ (Fekih et al., 2013), MutMap‐Gap (Takagi, Uemura, et al., 2013), and QTL‐Seq (Takagi, Abe, et al., 2013) to accommodate different scenarios. In maize, bulked segregant RNA‐Seq (BSR‐Seq) was introduced (S. Liu et al., 2012), and more detailed protocols for using BSA to clone maize mutant genes have been further developed (Best & McSteen, 2022; Klein et al., 2018).

Despite extensive research on mutant creation and gene mapping, the molecular mechanisms underlying maize kernel development remain complex and not fully understood. In this study, we used EMS mutagenesis on five inbred lines to create a large collection of maize kernel mutants. Using BSA with whole‐genome sequencing (WGS), we mapped the causative mutations for a shrunken kernel mutant and a small kernel mutant. Our results identified two key genes: the classic gene, ADP‐glucose transporter (*ZmBT1*, brittle endosperm 1), and the novel gene, DNA topoisomerase VI A subunit (*ZmTOP6A*).

## 2 MATERIALS AND METHODS

### 2.1 Maize materials and growth conditions

Five maize inbred lines were used in this study, all with similar flowering times (sown around May 1, flowering around July 25, and harvested around October 1). B73 and MO17 are widely used in the maize research community and have high‐quality genome assemblies. PH6WC and PH4CV are the parental lines of Xianyu335, one of the most popular commercial hybrids in China. W9816 is an elite inbred line from our breeding program, known for its good resistance to chilling stress. All lines were cultivated under regular field management practices in Changchun, China.

### 2.2 EMS mutagenesis of maize pollen

EMS mutagenesis of maize pollen was performed with slight modifications to previously established protocols (Neuffer & Coe, 1978; Settles, 2020). At flowering time, ears without emerged silks were bagged to prevent unintended fertilization. Fresh pollen was collected in the morning under favorable weather conditions, and anthers were discarded. A 10 mL volume of pollen was suspended in 100 mL of paraffin oil containing 0.1% (v/v) EMS. The mixture was used to pollinate the protected ears with a soft brush within 30 min. Ears were re‐bagged immediately after pollination to prevent contamination. Mutagenesis was carried out over 3–5 days to account for environmental variability. M_1_‐generation seeds were harvested and then propagated via self‐pollination. In the M_2_ generation, each ear was harvested individually and screened for kernel trait segregation.

### 2.3 DNA/RNA extraction, cDNA synthesis, and polymerase chain reaction amplification

Genomic DNA was extracted using a modified CTAB method (G. C. Allen et al., 2006). Total RNA was extracted using an Ultrapure RNA kit (CAT#: CW0597S, CWBIO). The quality and concentration of DNA and RNA samples were assessed using agarose gel electrophoresis and a NanoDrop One spectrophotometer (Thermo Fisher Scientific). First‐strand cDNA was synthesized using the UEIris II RT‐PCR System for First‐Strand cDNA Synthesis Kit (CAT#: R2028, US Everbright). High‐fidelity polymerase chain reaction (PCR) amplification was performed with PrimeSTAR GXL DNA Polymerase (CAT#: R050, Takara), while standard fidelity PCR for SNP (single‐nucleotide polymorphism) genotyping used TaKaRa Ex Taq (CAT#: RR001, Takara). Sanger sequencing was conducted by Comate Bioscience Co., Ltd., and the results were visualized using Chromas software and aligned via EMBOSS Needle (Madeira et al., 2024).

### 2.4 Gene mapping through BSA

For each mutant, two pools were created: a wild‐type pool (WP) and a mutant pool (MP), each containing over 30 kernels. Wild‐type kernels were germinated on filtered paper. After 3 days, seedlings were pooled in equal volumes. Mutant kernels, which did not germinate, were soaked in distilled water for 12 h, and embryos were manually dissected and pooled. Genomic DNA from both pools was subjected to WGS. Library preparation and sequencing were performed by Novogene on an Illumina Novaseq platform, generating 150 bp paired‐end reads. Clean reads were aligned to the maize Zm‐B73‐REFERENCE‐NAM‐5.0 genome (Hufford et al., 2021) using BWA (H. Li & Durbin, 2009). SAMtools and BCFtools (H. Li et al., 2009) were used for alignment processing and variant calling. Variant annotations were conducted using SnpEff (Cingolani et al., 2012), and GATK's “VariantsToTable” module (McKenna et al., 2010) was used to extract variant information, including chromosome, position, reference allele, alternative allele, genotype, and allelic depth.

**Core Ideas**

- Ethyl methanesulfonate mutagenesis of pollen was performed on five maize inbred lines, resulting in over 400 kernel mutants.
- ZmBT1 was identified as responsible for a shrunken kernel mutant, while ZmTOP6A was responsible for a small kernel mutant.
- The protein conservations, expression patterns, and subcellular localizations of ZmBT1 and ZmTOP6A were analyzed.

A modified “MutMap” approach (Abe et al., 2012) was used for association mapping. The SNP‐index, defined as the allele frequency of mutant‐type alleles, was calculated as follows: SNP‐index = (depth of alternative allele)/(total depth). The SNP‐index divergence (SNP‐index.DIV) was calculated to assess deviation from theoretical expectations of the causal mutation using the formula: SNP‐index.DIV = | SNP‐index.WP − theoretical SNP‐index.WP | + | SNP‐index.MP − theoretical SNP‐index.MP |. For a recessive mutation controlled by a single gene, theoretical SNP‐index values for the causal mutation in WP and MP are 1/3 and 1, respectively, and SNP‐index.DIV is 0. Candidate regions were identified based on the genome‐wide SNP‐index.DIV values, with markers showing SNP‐index.DIV < 1/3 is considered associated with the trait. Close inspection of SNP‐index.MP values and marker annotations helped pinpoint candidate causal mutations.

### 2.5 Genome assembly, gene annotation, and conservation analysis

The reference maize genome (Zm‐B73‐REFERENCE‐NAM‐5.0.55) was downloaded from Phytozome (https://phytozome‐next.jgi.doe.gov/). Gene structure and functional annotations were obtained from maizeGDB (https://maizegdb.org/), Uniprot (https://www.uniprot.org), and NCBI (https://www.ncbi.nlm.nih.gov). Gene structure was visualized using the Exon‐Intron Graphic Maker (http://www.wormweb.org/exonintron). Gene expression data across 22 maize tissues throughout development (Walley et al., 2016) were accessed via qTeller‐maizeGDB (Woodhouse et al., 2022). Homologs of ZmBT1 and ZmTOP6A were identified using OrthoDB v12 (Kuznetsov et al., 2023). Protein sequences were filtered by length (mean ± standard deviation), and multiple sequence alignment was conducted using Clustal‐Omega (Madeira et al., 2024). Principal component analysis (PCA) was performed using Jalview (Waterhouse et al., 2009).

### 2.6 Allelic verification

Additional EMS mutants were obtained from maizeEMSDB (https://maizeems.qlnu.edu.cn/), generated in the B73 background, and sequenced via exome‐capture (Lu et al., 2018). The *bt1‐3* mutant carries a stop‐gained mutation (c.352C > T|p.Q118*) in *ZmBT1* (Zm00001eb235570), and *top6a‐2* carries a stop‐gained mutation (c.1231C > T|p.Q411*) in *ZmTOP6A* (Zm00001eb013040). Genotypes of our own mutants and those from maizeEMSDB were confirmed by Sanger sequencing. Heterozygous plants were either self‐pollinated or crossed to assess allelism.

### 2.7 Subcellular localization in maize protoplasts

Full‐length coding sequences (excluding the stop codon) of target genes were cloned by reverse transcription polymerase chain reaction and inserted into the pSAT6‐EGFP‐N1 vector using a Ready‐to‐Use Seamless Cloning Kit (CAT#: B632219, Sangon). The resulting constructs expressed C‐terminal EGFP (enhanced green fluorescent protein) fusion proteins for subcellular localization. Maize seedlings were grown in the dark until the two‐leaf stage. Etiolated second leaves were cut into 1‐mm‐wide strips perpendicular to the veins and subjected to enzymatic digestion to isolate protoplasts. Plasmids (concentration > 500 ng/µL) were introduced into protoplasts using the PEG/Ca^2^^+^ transformation method (Zhai et al., 2009). After 12 h of incubation at room temperature in the dark, fluorescence was observed using a Leica STELLARIS 5 confocal microscope.

## 3 RESULTS

### 3.1 Quick creation of maize kernel mutants via EMS mutagenesis of pollen

**FIGURE 1:**
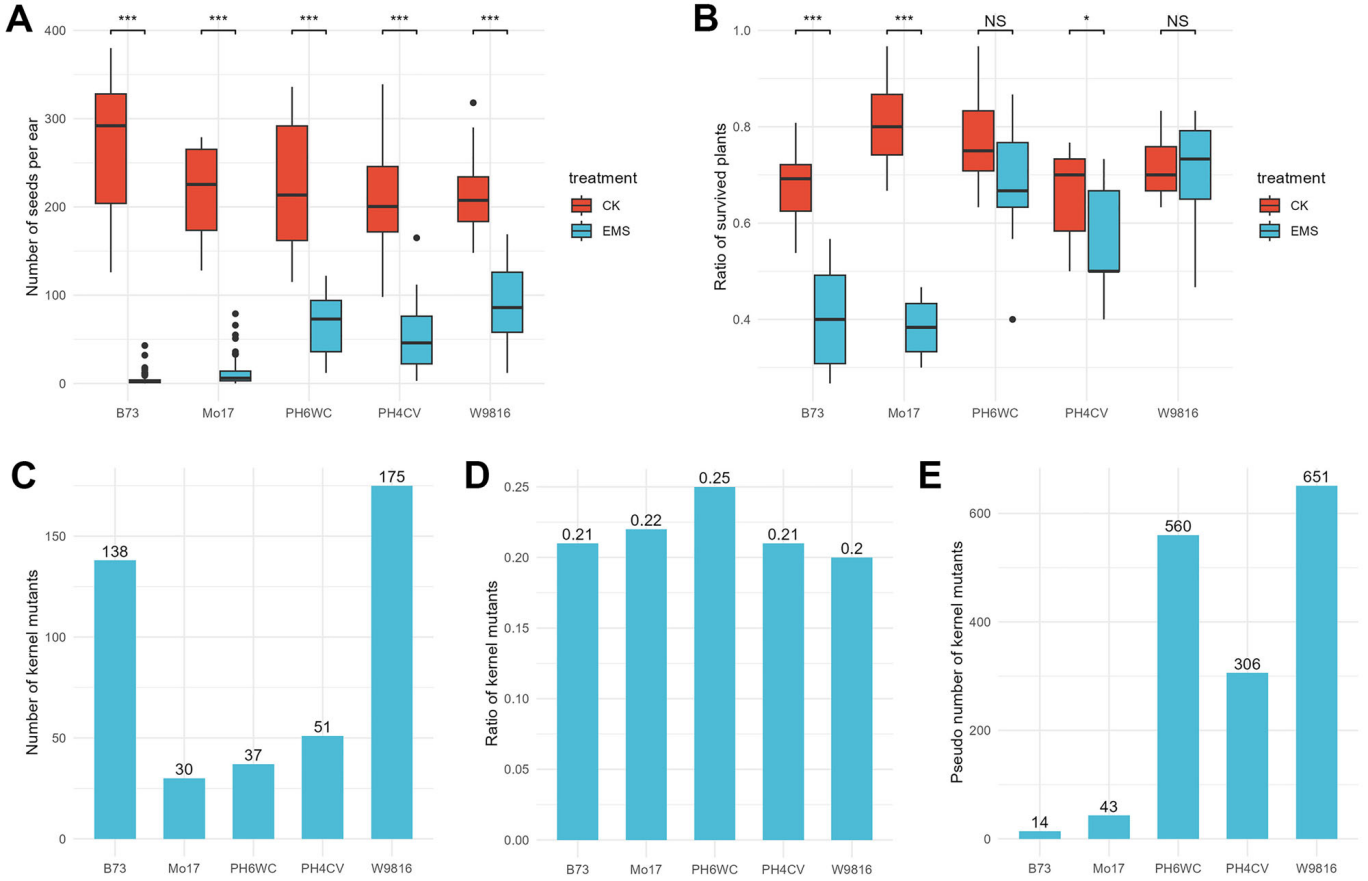
Performance of maize inbred lines after ethyl methanesulfonate (EMS) mutagenesis of pollen. (A) Number of seeds per ear after mutagenesis. Data for the control were based on over 10 ears with normal pollination, while data for EMS were based on over 30 ears after EMS mutagenesis of pollen. (B) Survival ratios of plants. Data were based on 10 rows, each containing 30 seeds. Statistical significance was determined by a *t*‐test: *p‐value < 0.05; **p‐value < 0.01; ***p‐value < 0.001; NS, not significant. (C) Number of kernel mutants obtained for each inbred line. (D) Ratio of kernel mutants to the total number of harvested ears. (E) Estimated number of kernel mutants generated from 100 ears following EMS mutagenesis of pollen. CK, control.

Five maize inbred lines (B73, MO17, PH6WC, PH4CV, and W9816), which all exhibit similar flowering times, were subjected to EMS mutagenesis of pollen. The mutagenesis resulted in varying performances across the lines (Figure 1A; Figure S1). The number of kernels per ear in the M_1_ generation was significantly reduced compared to the control (normal pollination). B73 and MO17 were the most severely affected, while W9816 was the least impacted. Specifically, B73 and MO17 produced only 3.3 and 10.5 seeds per ear, respectively, while PH6WC, PH4CV, and W9816 were able to produce 50–100 seeds per ear. M_1_ seeds were then planted in the field and propagated via self‐pollination. The survival ratios of B73 and MO17 plants post‐EMS mutagenesis dropped to 40%, about half the survival rate of the control group. In contrast, the other three lines maintained survival ratios of 60% or higher, with no significant differences from the control (Figure 1B).

In total, 421 maize kernel mutants (M_2_ generation) were obtained, with 128 from the B73 background, 30 from MO17, 37 from PH6WC, 51 from PH4CV, and 175 from W9816 (Figure 1C). The mutant ratios across all five lines were approximately 20% (Figure 1D). The different total numbers of kernel mutants among inbred lines mainly resulted from the unequal numbers of M_1_ seeds planted. For example, more M_1_ seeds were planted for B73 and W9816, resulting in more kernel mutants despite similar mutant ratios across lines. Assuming that half of the surviving plants could be successfully self‐pollinated, we estimated the number of kernel mutants generated from 100 ears following EMS mutagenesis using the formula: 100 × number of seeds per ear × survival ratio × 1/2 (pollination success) × kernel mutant ratio. The estimated number of kernel mutants per 100 ears was 14 for B73, 43 for MO17, 560 for PH6WC, 306 for PH4CV, and 651 for W9816 (Figure 1E). These results suggest that modern commercial inbred lines (PH6WC, PH4CV, and W9816) are more suitable for generating maize kernel mutants.

**FIGURE 2:**
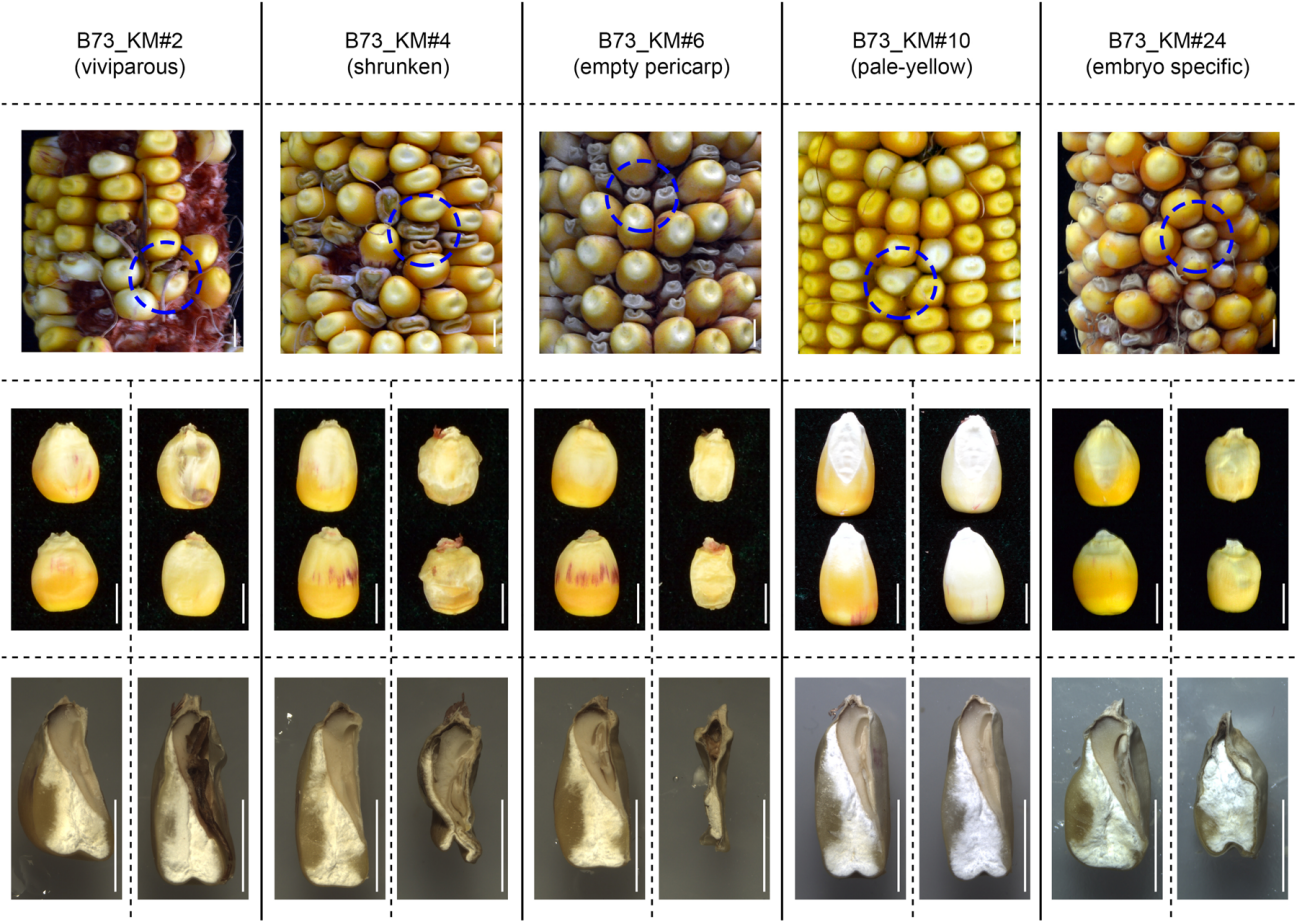
Representative maize kernel mutants in the B73 background. B73_KM#2 is a viviparous mutant with a pale‐yellow color, #4 is a shrunken mutant with almost no endosperm, #6 is an empty pericarp mutant with minimal embryo or endosperm, #10 is a pale‐yellow mutant, and #24 is an embryo‐specific mutant with no embryo. For each mutant, the left panel shows a wild‐type kernel, and the right panel shows a mutant kernel. Scale bar = 0.5 cm.

The kernel mutants displayed a range of mutation types, with varying degrees of defectiveness (Figure 2; Figure S2). For instance, B73_KM#2 is a viviparous mutant with a pale‐yellow color, B73_KM#4 is a shrunken mutant with nearly no endosperm, B73_KM#6 is an empty pericarp mutant with almost no embryo or endosperm, B73_KM#10 is a pale‐yellow mutant, and B73_KM#24 is an embryo‐specific mutant with no embryo. Moreover, most of the mutants segregated in a 3:1 ratio of wild‐type to mutant kernels, indicating that recessive alleles of single genes control the mutant phenotypes. Detailed figures for all maize kernel mutants have been deposited in a public data repository (see Data Availability Statement). These include images showing ears with segregated kernels, representative kernel images, and longitudinal sections of the kernels. These materials will be invaluable for studying maize kernel development.

### 3.2 Phenotypic observation and gene mapping of the shrunken kernel mutant (B73_KM#4)

**FIGURE 3:**
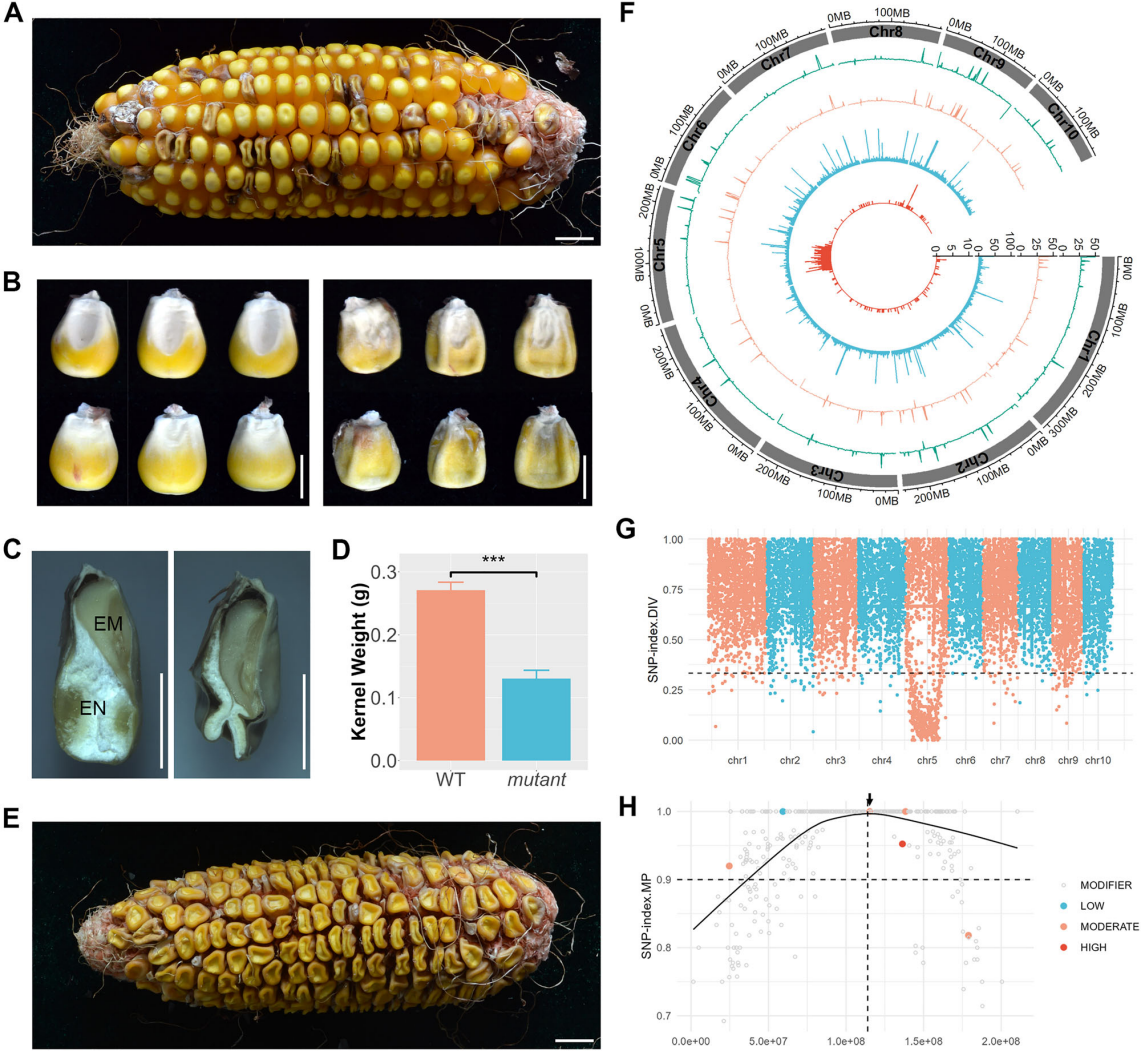
Phenotypic observation and gene mapping of the shrunken kernel mutant (B73KM#4). (A) Ear showing segregation of wild‐type (WT) and shrunken kernels. Scale bar = 1 cm. (B) Two side views of representative kernels. Scale bar = 0.5 cm. (C) Longitudinal section of representative kernels. EM, embryo; EN, endosperm. Scale bar = 0.5 cm. (D) Comparison of kernel weight. Data represent over 20 kernels (biological replicates) and are shown as mean ± SD (standard deviation). Statistical significance was determined by a *t*‐test: ***p‐value < 0.001. (E) An ear from the self‐pollination of mutant kernels. Scale bar = 1 cm. (F) Circos plot showing whole‐genome sequencing data. The outer two tracks represent sequencing depths for the wild‐type pool (WP) and mutant pool (MP). The inner two tracks display the distribution of detected single‐nucleotide polymorphisms (SNPs) and filtered SNPs. The window size is 1 MB. (G) Genome‐wide view of SNP‐index.DIV. The horizontal dashed line marks the one‐third cutoff for filtering markers. (H) Close‐up view of SNP‐index.MP and annotations for filtered markers on chromosome 5. The black curve represents smoothed values, and the dashed vertical line indicates the peak of these values.

B73_KM#4 (*bt1‐1*) exhibits a classic shrunken/collapsed kernel phenotype (Figure 3A,B). The endosperm in the mutant kernels was nearly absent (Figure 3C), and the kernel weight was significantly reduced, approximately 50% of wild‐type kernels (Figure 3D). Despite the minimal residual endosperm providing some nutrients, some mutant kernels were still capable of germination, growing into plants, and completing pollination, resulting in ears filled with mutant kernels (Figure 3E).

To map the causal gene, we employed the BSA strategy. WGS generated 421.8 million clean reads (63.3 Gb) for the WP and 432.0 million clean reads (64.8 Gb) for the MP, achieving average sequencing depths of 28.4× and 29.5×, respectively. A total of 23,842 SNPs were detected, and 478 SNPs (SNP‐index.DIV < 1/3) were filtered as potential markers associated with the causal mutation. Although the distribution of the detected SNPs across the genome was relatively uniform, the filtered SNPs were notably enriched on chromosome 5 (Figure 3F; Figure S3). A genome‐wide view of SNP‐index.DIV revealed a clear association of the causal mutation on chromosome 5 (Figure 3G). Closer inspection of SNP‐index.MP and annotations for the filtered SNPs led to the identification of a missense mutation (c.464C > T|p.P155L) in the gene Zm00001eb235570 (*brittle endosperm 1*, *ZmBT1*), which was homozygous in the MP (Figure 3H).

**FIGURE 4:**
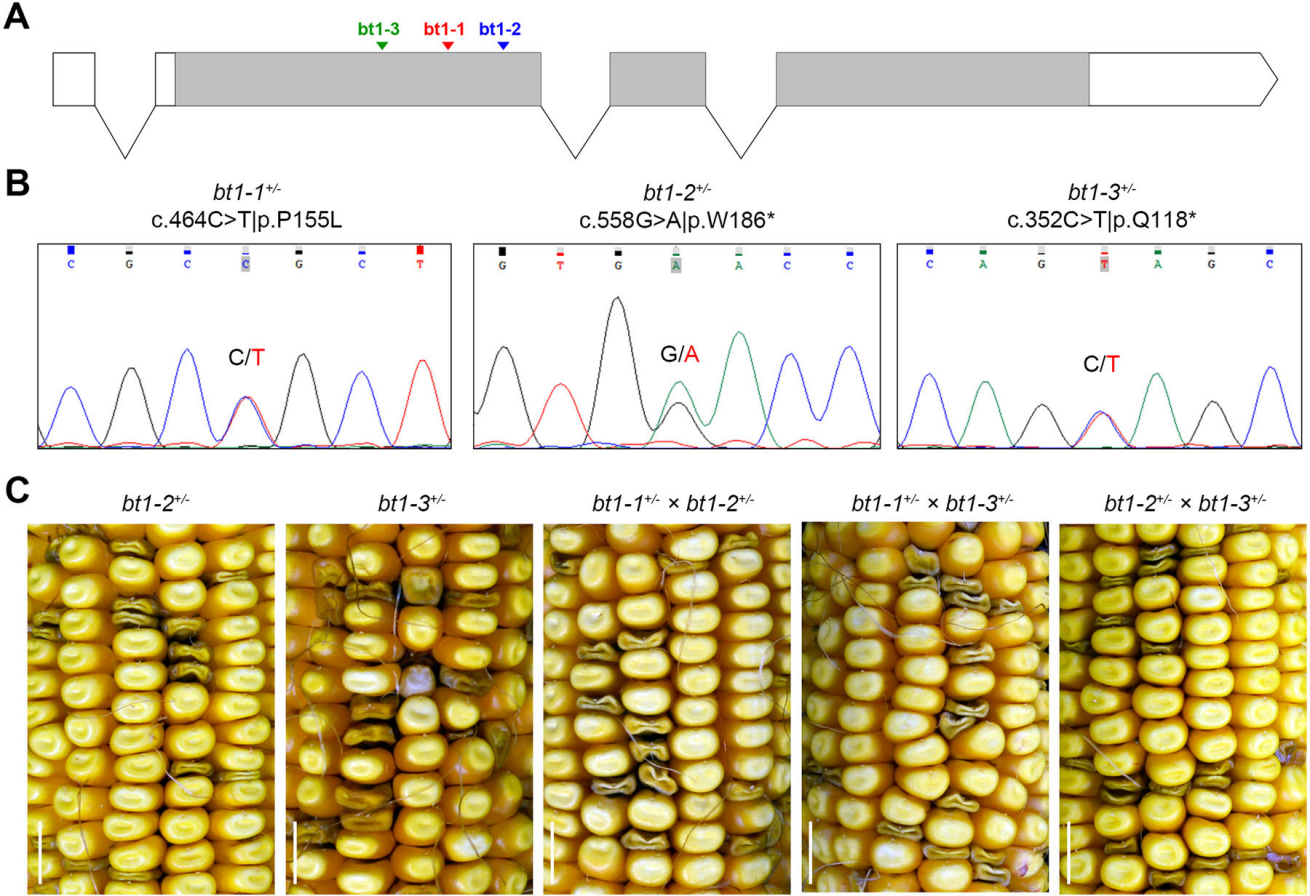
Allelic verification for *ZmBT1*. (A) Gene structure and mutation locations for *ZmBT1*: *bt1‐1* (c.464C > T|p.P155L), *bt1‐2* (c.558G > A|p.W186*), and *bt1‐3* (c.352C > T|p.Q118*). (B) Sanger sequencing of heterozygotes for the three mutation alleles of *ZmBT1*. (C) Ears from self‐pollination or cross‐pollination of the heterozygotes. Scale bar = 1 cm.

*ZmBT1* is a well‐characterized maize gene (Schnable & Freeling, 2011) encoding a 437‐amino acid protein, an ADP‐glucose transporter involved in maize endosperm starch biosynthesis. Mutations in *ZmBT1* are known to lead to brittle endosperm and the shrunken kernel phenotype (Kirchberger et al., 2007; Shannon et al., 1998; Sullivan & Kaneko, 1995). To further confirm that the missense mutation in *ZmBT1* was responsible for the shrunken kernel phenotype in B73_KM#4, we performed allelic tests. A stop‐gained mutation (c.558G > A|p.W186*) was further identified in another shrunken kernel mutant (B73_KM#9, *bt1‐2*), and a third mutant (*bt1‐3*) from maizeEMSDB carries a c.352C > T|p.Q118* mutation (Figure 4A). Heterozygotes for all three mutations *(bt1‐1^+/−^*, *bt1‐2^+/−^*, *bt1‐3^+/−^*) were identified and crossed with each other (Figure 4B). Self‐crosses and crosses of these heterozygotes (*bt1‐1^+/−^* × *bt1‐2^+/−^*, *bt1‐1^+/−^* × *bt1‐3^+/−^*, *bt1‐2^+/−^* × *bt1‐3^+/−^*) all exhibited similar segregation patterns of wild‐type and shrunken kernels (Figure 4C). Taken together, the gene mapping and allelic verification established that mutations in *ZmBT1* are responsible for the shrunken kernel phenotype in *bt1‐1/2/3*.

### 3.3 Conservation analysis, gene expression pattern, and subcellular localization of ZmBT1

**FIGURE 5:**
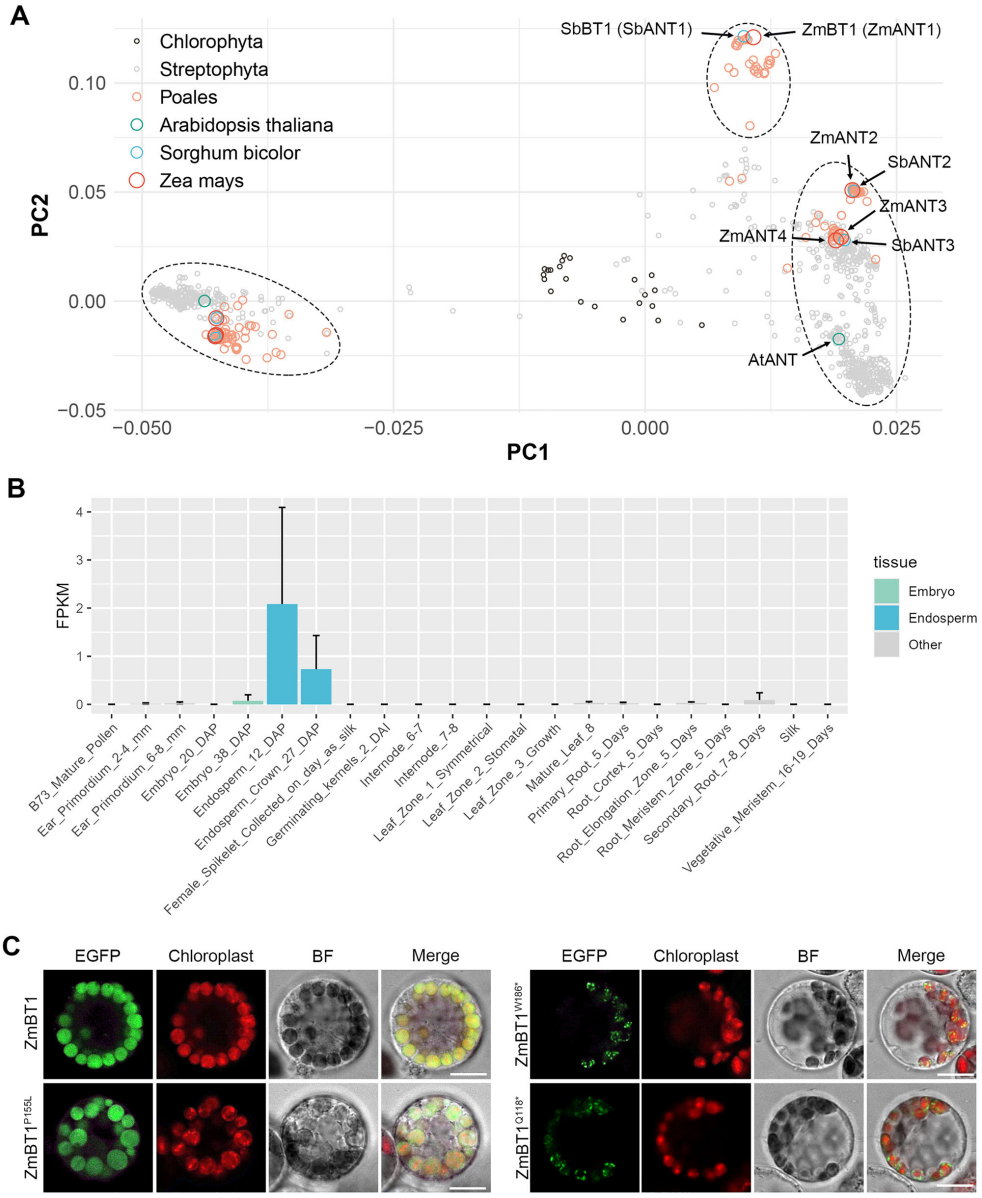
Conservation analysis, gene expression pattern, and subcellular localization of ZmBT1. (A) Conservation analysis of ZmBT1 and its homologs. Multiple sequence alignment and principal component analysis (PCA) were performed on 1071 proteins from 301 plant species in the “adenine nucleotide transporter BT1” group. (B) Expression pattern of *ZmBT1* across 22 maize tissues. Data are obtained from qTeller‐maizeGDB. (C) Subcellular localization of ZmBT1 and its mutant variants. Enhanced green fluorescent protein (EGFP)‐tagged constructs were transiently expressed in maize leaf protoplasts. Chloroplasts were visualized by autofluorescence. BF, bright field; FPKM, fragments per kilobase of transcript per million mapped reads. Scale bar = 10 µm.

To further extend our understanding of *ZmBT1*, we performed functional annotation of this gene. According to the OrthoDB database, ZmBT1 belongs to the “adenine nucleotide transporter BT1” family (Group 1526107at33090 at the Viridiplantae level). To explore its evolutionary conservation, we performed multiple sequence alignment and PCA on 1071 proteins from 301 species within this group (Figure 5A). ZmBT1 (also referred to as ZmANT1) clustered with a small subgroup of proteins specific to Poales species. In maize, there are three close paralogs of ZmBT1 (ZmANT2, ZmANT3, and ZmANT4), while Arabidopsis encodes a closely related ortholog (AtANT). These proteins form a second cluster conserved across both Poales and non‐Poales species. A third, more divergent cluster contains ancient homologs of ZmBT1, suggesting an early origin of this gene family.

We next examined the gene expression pattern and subcellular localization of ZmBT1. Transcriptome analysis revealed that *ZmBT1* is specifically and highly expressed in the developing endosperm (Figure 5B), consistent with its role in starch biosynthesis. To determine subcellular localization, we expressed EGFP‐tagged versions of ZmBT1 and its mutant forms (*bt1‐1*: ZmBT1^P155L^, *bt1‐2*: ZmBT1^W186*^, *bt1‐3*: ZmBT1^Q118*^) in maize leaf protoplasts. All constructs localized to chloroplasts, as visualized by chlorophyll autofluorescence. Interestingly, while ZmBT1 and ZmBT1^P155L^ displayed a diffuse, even distribution within the chloroplast, ZmBT1W186* and ZmBT1^Q118*^ showed a punctate, dotted pattern around the chloroplast (Figure 5C). Given that endosperm cells lack chloroplasts, the equivalent organelle in these cells is the amyloplast. Thus, ZmBT1 functions within amyloplasts of maize endosperm, consistent with its role in transporting ADP‐glucose for starch biosynthesis.

### 3.4 Phenotypic observation and gene mapping of a novel small kernel mutant (B73KM#44)

**FIGURE 6:**
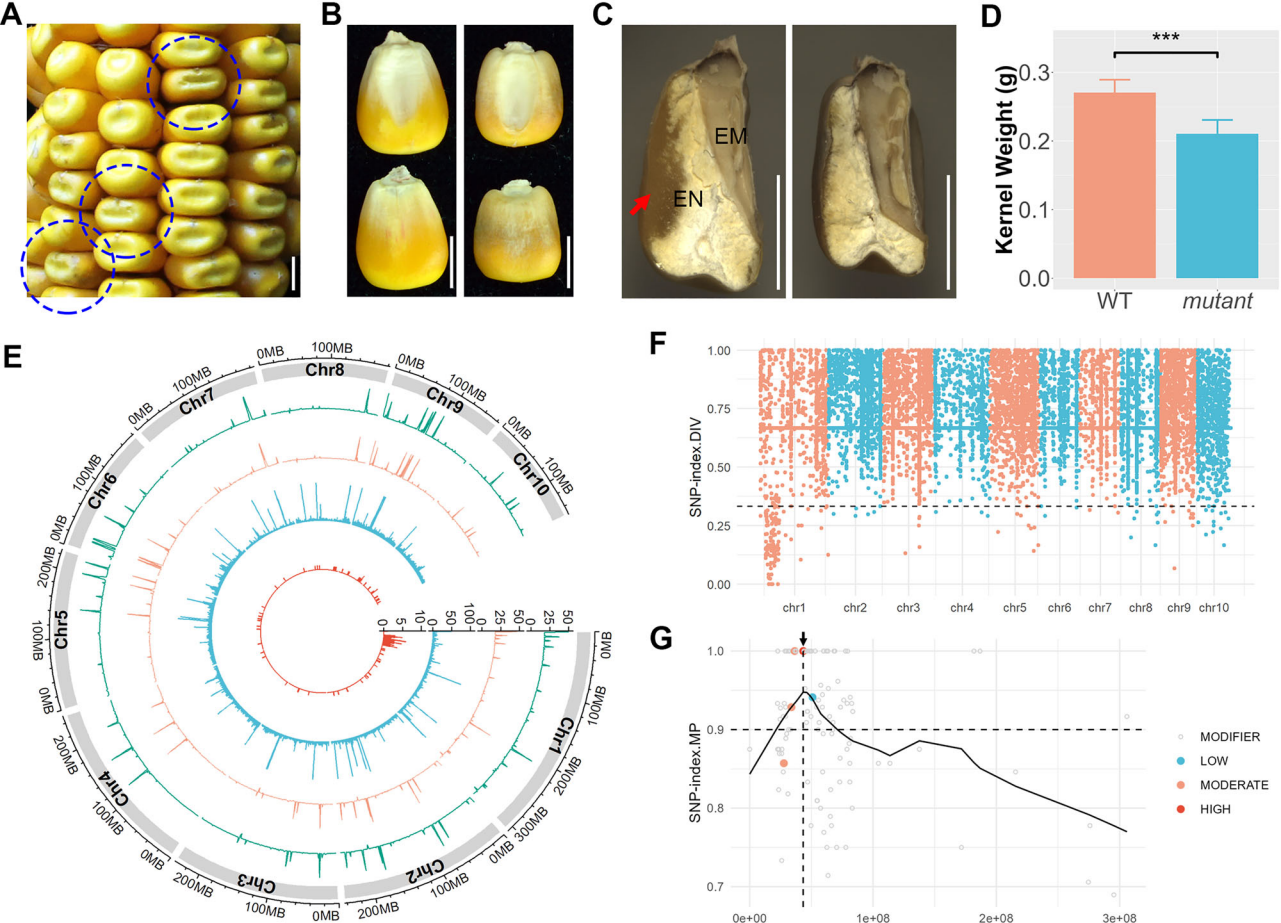
Phenotypic observation and gene mapping of a small kernel mutant (B73KM#44). (A) Ear showing segregation of wild‐type (WT) and mutant kernels. Scale bar = 0.5 cm. (B) Two‐sided views of kernels. Scale bar = 0.5 cm. (C) Longitudinal section of kernels. EM, embryo; EN, endosperm. The red arrow points horny‐type endosperm. Scale bar = 0.5 cm. (D) Comparison of kernel weight. Data are based on over 20 kernels (biological replicates) and are presented as mean ± SD (standard deviation). Significance was determined using a *t*‐test: ***p‐value < 0.001. (E) Circos plot showing whole‐genome sequencing data. The outer two tracks display sequencing depth for the wild‐type pool (WP) and mutant pool (MP), while the inner two tracks display the distribution of detected and filtered single‐nucleotide polymorphisms (SNPs). The window size is 1 MB. (F) Genome‐wide view of SNP‐index.DIV, with the horizontal dashed line indicating the one‐third cutoff for filtering markers. (G) Close‐up view of SNP‐index.MP and annotations for filtered markers on chromosome 1. The black curve represents smoothing values, and the dashed vertical line indicates the peak of these values.

In addition to investigating well‐known kernel mutants and their associated genes, we aimed to identify novel kernel mutants and genes. B73_KM#44 is a newly discovered small (or defective) kernel mutant (Figure 6A,B). The typical horny‐type endosperm is absent in this mutant (Figure 6C), and the kernel weight is approximately 20% lower than that of wild‐type kernels (Figure 6D). In addition, the mutant kernels are nonviable and fail to germinate, for reasons that remain unclear.

For gene mapping, WGS generated 352.1 million and 351.0 clean reads, corresponding to 52.8 and 52.7 Gbs for the WP and MP, respectively. The average sequencing depths were 19.7× for WP and 18.3× for MP. A total of 19,033 SNPs were detected, distributed evenly across the genome. Of these, 168 filtered SNPs (with SNP‐index.DIV < 1/3) were notably enriched on chromosome 1 (Figure 6E; Figure S4). A genome‐wide view of the SNP‐index.DIV indicated that the causal mutation lies near the start of chromosome 1 (Figure 6F). Further examination of filtered markers identified two candidate mutations (Figure 6G). Both mutations were 100% homozygous in the MP and were predicted to have MODERATE/HIGH impacts. One mutation was a start‐lost mutation (c.3G > A|p.M1?) in gene *Zm00001eb013040* (DNA topoisomerase VI A subunit, *ZmTOP6A*), while the other was a missense mutation (c.203A > T|p.D68V) in gene *Zm00001eb011300* (ERAD‐associated E3 ubiquitin‐protein ligase component HRD3A).

**FIGURE 7:**
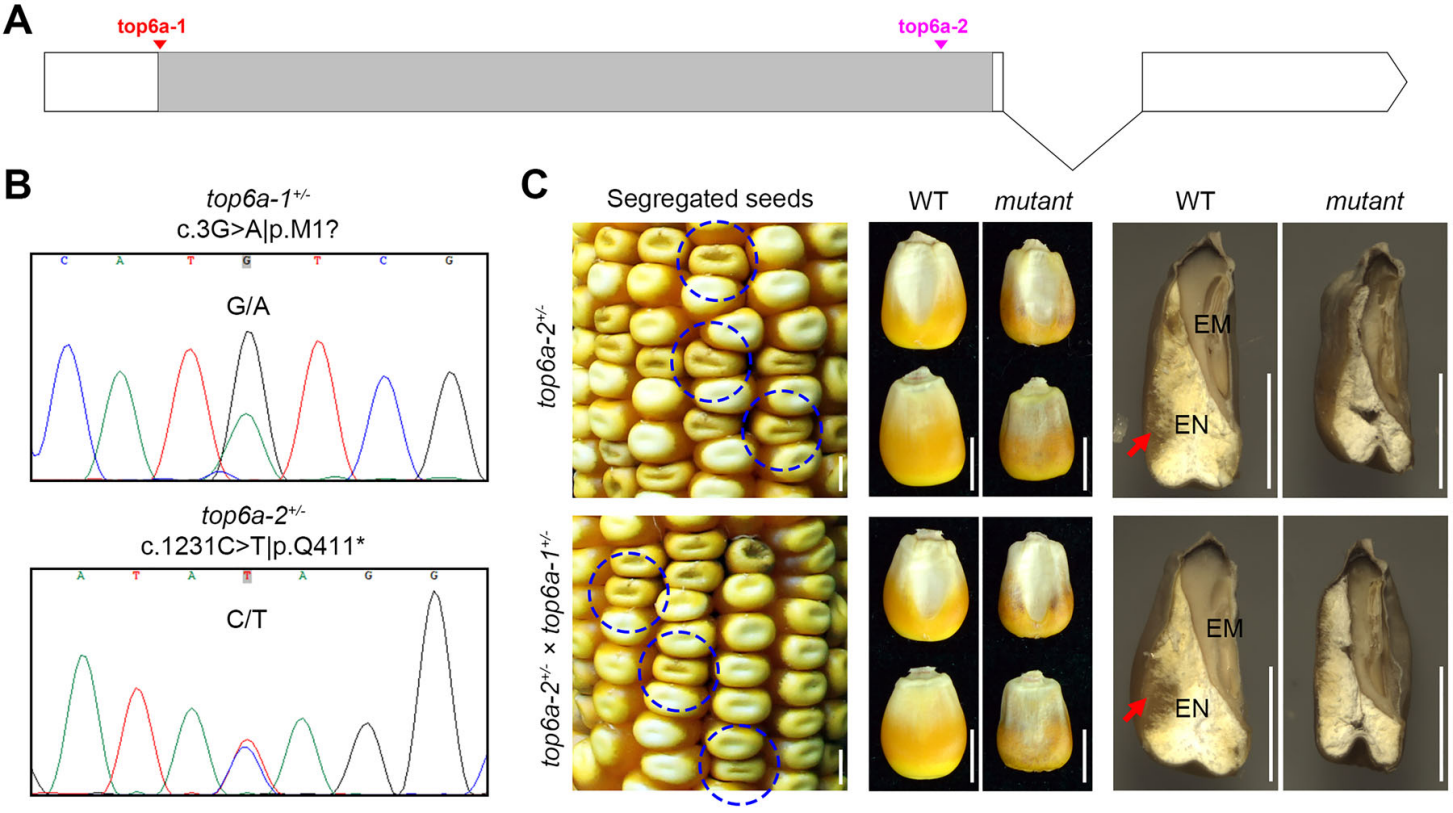
Allelic validation for *ZmTOP6A*. (A) Gene structure and mutation locations for *ZmTOP6A*: *top6a‐1* (c.3G > A|p.M1?) and *top6a‐2* (c.1231C > T|p.Q411*). (B) Sanger sequencing of heterozygotes for the two mutation alleles of *ZmTOP6A*. (C) Ears and seeds from self‐pollination or cross‐pollination of heterozygotes. EM, embryo; EN, endosperm; WT, wild‐type. The red arrows point horny‐type endosperm. Scale bar = 0.5 cm.

The start‐lost mutation in *ZmTOP6A* is expected to result in a loss‐of‐function allele, whereas the missense mutation in *ZmHRD3A* is less likely to affect gene function. Based on this, we hypothesize that the start‐lost mutation in *ZmTOP6A* is the likely cause of the small kernel phenotype. To test this, we obtained a second EMS‐induced mutant (*top6a‐2*) for *ZmTOP6A* from maizeEMSDB, which harbors a stop‐gained mutation (c.1231C > T|p.Q411*) (Figure 7A). Heterozygous individuals were identified for both *top6a‐1* (B73_KM#44) and *top6a‐2* alleles by Sanger sequencing (Figure 7B). These heterozygous plants were self‐ and cross‐pollinated, and the resulting offspring exhibited a segregation pattern of wild‐type and mutant kernels. The mutant kernels displayed a phenotype similar to that of B73_KM#44 (Figure 7C). Together, the gene mapping and allelic test results confirm that loss‐of‐function mutations in *ZmTOP6A* are responsible for the small kernel phenotype, characterized by smaller size, absence of horny endosperm, and seed lethality.

### 3.5 Conservation analysis, gene expression pattern, and subcellular localization of ZmTOP6A

**FIGURE 8:**
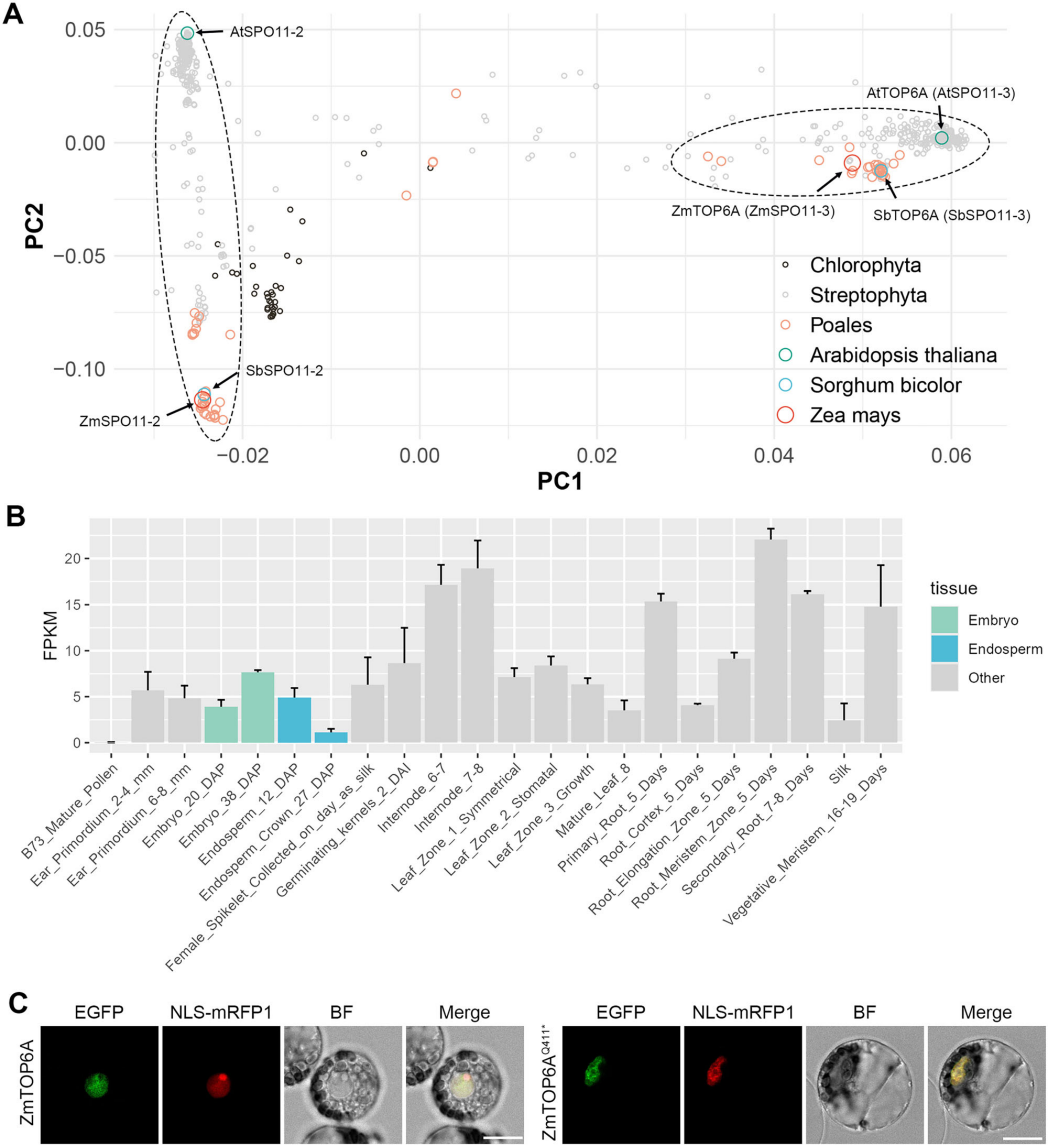
Conservation analysis, gene expression pattern, and subcellular localization for ZmTOP6A. (A) Conservation analysis of ZmTOP6A and its homologs. Multiple sequence alignment and principal component analysis (PCA) were conducted on 612 proteins from 308 plant species within the “topoisomerases” group. (B) Spatial expression pattern of *ZmTOP6A* based on RNA‐Seq data spanning 22 maize tissues. Data were retrieved from qTeller‐maizeGDB. (C) Subcellular localization of ZmTOP6A and its mutant forms. The target protein was fused with an enhanced green fluorescent protein (EGFP) tag at its C‐terminus, and nuclear localization signal (NLS)‐mRFP1 was used as a marker. BF, bright field. Scale bar = 10 µm.

To further explore the function of *ZmTOP6A*, we conducted a series of analyses. ZmTOP6A is a member of the “topoisomerases” gene family (Group 1405863at33090 at the Viridiplantae level, OrthoDB v12). Multiple sequence alignments and PCA were performed on 612 proteins from 308 plant species (Figure 8A). ZmTOP6A, along with its close homologs in sorghum (*Sorghum bicolor* (L.) Moench) (SbSPO11‐3) and Arabidopsis (AtTOP6A/AtSPO11‐3), formed one distinct cluster. Their ancient paralogs (ZmSPO11‐2, SbSPO11‐2, AtSPO11‐2) grouped into a second and more distant cluster. The spatial expression pattern of *ZmTOP6A* showed no clear tissue specificity, suggesting that it may function throughout the entire maize life cycle (Figure 8B). This aligns with the lethal phenotype observed in the *top6a* mutant. Subcellular localization analysis revealed that both ZmTOP6A and its mutant form (ZmTOP6A^Q411*^) localized to the nucleus, consistent with its role as a DNA topoisomerase (Figure 8C).

## 4 DISCUSSION

### 4.1 Rapid creation of maize kernel mutants using EMS mutagenesis of pollen

In this study, we demonstrated the high efficiency of using EMS mutagenesis of pollen to generate maize kernel mutants. Previous studies have primarily focused on a single maize inbred line, showing that EMS mutagenesis can effectively generate mutants (Chong et al., 2025; Lu et al., 2018; Nie et al., 2021; Tran et al., 2020). However, none of these studies have compared multiple lines within a single experiment or focused on the detailed characterization of kernel mutants. In our study, five representative inbred lines were selected and subjected to EMS mutagenesis, each displaying distinct performances after treatment (Figure 1). Our predictions suggest that, starting with 100 ears, B73 and MO17 would produce only 13 and 37 kernel mutants, while the three modern commercial lines could generate 300–600 kernel mutants. These results clearly demonstrate that the modern commercial maize inbred lines are more efficient at generating kernel mutants than classical inbred lines. Additionally, utilizing multiple inbred lines allows us to capture a broader spectrum of genetic and phenotypic variations, which may be both line‐specific and conserved across different genetic backgrounds. Over two growth seasons (the first for EMS mutagenesis and the second for propagation), we successfully obtained approximately 400 independent kernel mutants (M_2_‐generation). These mutants exhibited a wide range of phenotypic variations, including various defects in the embryo, endosperm, and other kernel structures (Figure 2; Figure S2).

### 4.2 Advantages of combining EMS mutagenesis and BSA for gene mapping

The BSA strategy was successfully employed to map two representative kernel mutants, leading to the identification of one classical gene (*ZmBT1*) and a novel gene (*ZmTOP6A*). This highlights the effectiveness and advantages of combining EMS mutagenesis with BSA for gene mapping in maize. EMS mutagenesis generates a broad range of SNPs that are randomly distributed across the genome. These SNPs can be easily detected using current high‐throughput sequencing, making them excellent markers for gene mapping using BSA. This approach eliminates the need to cross the mutant with a different ecotype, which could introduce unwanted effects like heterosis, complicating the identification of the target phenotype and hindering the distinction of the causal mutation (Abe et al., 2012).

With the rapid advancement of high‐throughput sequencing technologies, the cost of sequencing has dropped significantly. Looking ahead, the creation and mapping of maize EMS‐induced kernel mutants will become increasingly feasible. This will facilitate the identification of numerous genes involved in maize kernel development, contributing to a deeper understanding of the genetic network that underpins kernel traits. With this knowledge, breeders will be able to precisely select and combine favorable genes to develop desirable maize varieties.

### 4.3 The involvement of ZmBT1 in maize storage starch biosynthesis

*ZmBT1* (ADP‐glucose transporter) was identified and confirmed as the causal gene for the shrunken kernel mutants, which exhibited severe defects in the endosperm development (Figures 3 and 4). *BT1* is a well‐characterized gene in starch biosynthesis in cereal endosperm (C. Li et al., 2018). In this process, sucrose is produced in photosynthetic tissues and transported to the developing endosperm. In the cytosol of endosperm cells, sucrose is converted into ADP‐glucose, which is then transported into the amyloplast by ZmBT1 (Kirchberger et al., 2007). Inside the amyloplast, ADP‐glucose follows two pathways: one leads to the amylose formation via granule‐bound starch synthase, and the other leads to amylopectin synthesis (Figure S5). Loss‐of‐function in ZmBT1 impairs this transport step, blocking starch biosynthesis and resulting in the accumulation of free sugars that are not incorporated into starch granules.

Our conservation analysis revealed that ZmBT1 belongs to a gene cluster unique to Poales (Figure 5A). Expression profiling and subcellular localization confirmed that ZmBT1 is specifically expressed in maize endosperm and localizes to the amyloplast (Figure 5B,C). Knockout or knockdown of *BT1* genes in other Poales species such as rice, wheat (*Triticum aestivum* L.), and barley (*Hordeum vulgare* L.) results in similar shrunken kernel phenotypes, accompanied by reduced starch content (Cakir et al., 2016; H. Liu et al., 2023; Wang et al., 2019). In contrast, non‐Poales species, lacking ZmBT1‐like transporter, may use alternative pathways in which glucose‐6‐phosphate or glucose‐1‐phosphate enters the amyloplast and is converted into ADP‐glucose within the plasmid (Tetlow, 2011). Therefore, *BT1* may have played a key role in the evolutionary divergence of Poale, contributing to their efficient starch biosynthesis and making them well‐suited as staple grains.

ZmBT1 has three paralogs (ZmANT2‐4) that share a common Arabidopsis ortholog (AtANT), and are found in a gene cluster conserved across Poales and non‐Poales species. Unlike *ZmBT1*, which is endosperm‐specific, *ZmANT2‐4* are consistently expressed across all maize tissues, except mature pollen (Figure S6). Previous studies have shown that *AtANT* mutants in Arabidopsis exhibit growth abnormalities and sterility (Kirchberger et al., 2008). AtANT is dually localized to plastids and mitochondria, acting as a nucleotide uniport carrier that exports adenylates into the cytosol (Bahaji et al., 2011). While ZmBT1 is specialized for endosperm starch biosynthesis, its paralogs ZmANT2‐4 may fulfill housekeeping roles in nucleotide transport across plastid/mitochondrial membranes (consistent with their ubiquitous expression), which requires further functional validation.

### 4.4 The involvement of ZmTOP6A in maize kernel development

*ZmTOP6A* (also known as *ZmSPO11‐3*) was identified and validated as the causal gene underlying a small‐kernel mutant that leads to lethality. This gene encodes the A subunit of DNA topoisomerase VI (Topo VI), an enzyme complex essential for managing DNA topology. During transcription and replication, the double helix must unwind, generating torsional stress that requires relief through topological rearrangements. DNA topoisomerases fulfill this role by transiently cleaving DNA and rejoining it (McKie et al., 2021). Topoisomerases are broadly classified into type I, which induce transient single‐strand breaks, and type II, which induce double‐strand breaks (Corbett & Berger, 2003). Within type II enzymes, a distinct subclass termed IIB was first identified in archaea and is now known as Topo VI (Bergerat et al., 1997, 1994). In prokaryotes, Topo VI forms an A_2_B_2_ heterotetramer composed of two A and two B subunits. In eukaryotes, Topo VI contains two A and B subunits, together with two small accessory subunits, RHL1 and BIN4, that stabilize the complex (Brinkmeier et al., 2022).

In plants, SPO11‐1 and SPO11‐2, which are homologous to TOP6A/SPO11‐3, are essential for meiotic double‐strand break formation and are not functionally redundant with TOP6A (A. M. Allen & Maxwell, 2024). Arabidopsis *spo11‐1* and *spo11‐2* single mutants resemble wild‐type plants, whereas *spo11‐3* (*top6a*) mutants exhibit severe developmental defects, including chlorotic cotyledons, small siliques, and other growth abnormalities (Šimková et al., 2012; Yin et al., 2002). In rice, *top6a* and *top6b* mutants show significant developmental delays and stunted growth (Xu et al., 2023). Consistent with these findings, maize *top6a* mutants exhibit abnormal kernel structure and lethality. This highlights the essential role of the Topo VI complex in plant growth and development, and warrants further study to fully unravel the underlying mechanisms.

## AUTHOR CONTRIBUTIONS

**Haixiao Dong**: Conceptualization; data curation; formal analysis; funding acquisition; investigation; methodology; software; visualization; writing—original draft. **Hao Chen**: Data curation; formal analysis; investigation; methodology; visualization; writing—original draft. **Yuan Jiang**: Data curation; investigation; resources; supervision. **Jingzhe Zhang**: Data curation; investigation. **Chaoyue Wang**: Data curation; investigation. **Zhili Sun**: Data curation; investigation. **Shengzhong Su**: Resources; supervision; validation. **Shipeng Li**: Resources; supervision; validation. **Hongkui Liu**: Resources; supervision. **Xiaohui Shan**: Conceptualization; funding acquisition; methodology; resources; supervision; validation; writing—review and editing. **Yaping Yuan**: Conceptualization; funding acquisition; project administration; resources; supervision; validation; writing—review and editing.

## CONFLICT OF INTEREST STATEMENT

The authors declare no conflicts of interest.

## Supporting information



Figure S1. Harvested ears for different maize inbred lines after EMS mutagenesis of pollen.
Figure S2. Representative kernel mutants in the W9816 background with varying defectiveness
Figure S3. Distributions and types of detected and filtered SNPs for gene mapping of B73_KM#4
Figure S4. Distribution and types of detected and filtered SNPs for gene mapping of B73_KM#44
Figure S5. The involvement of ZmBT1 in maize endosperm starch biosynthesis
Figure S6. The spatial expression patterns of ZmANT2, ZmANT3, and ZmANT4

## Data Availability

The WGS data for bulked segregant analysis (BSA) of B73_KM#4 (ZmBT1‐P155L) and B73_KM#44 (ZmTOP6A‐M1?) are available at NCBI‐SRA (PRJNA1227007). Figures for all generated maize kernel mutants, the BSA gene mapping pipeline, and detailed information of SNPs identified for B73_KM#4 and B73_KM#44 are available at Dyrad (https://doi.org/10.5061/dryad.h9w0vt4xb).
