# Supplementary material for: Quick creation and mapping of EMS‐induced maize kernel mutants identifies classical gene ZmBT1 and novel gene ZmTOP6A
Source: Plant Genome. 2026 Feb 23;19(1):e70210. doi: 10.1002/tpg2.70210 (PMC12927126; doi:10.1002/tpg2.70210)
Supplement: Supplementary file 1 — Figure S1. Harvested ears for different maize inbred lines after EMS mutagenesis of pollen. Figure S2. Representative kernel mutants in the W9816 background with varying defectiveness Figure S3. Distributions and types of detected and filtered SNPs for gene mapping of B73_KM#4 Figure S4. Distribution and types of detected and filtered SNPs for gene mapping of B73_KM#44 Figure S5. The involvement of ZmBT1 in maize endosperm starch biosynthesis Figure S6. The spatial expression patterns of ZmANT2, ZmANT3, and ZmANT4 [file TPG2-19-e70210-s001.pdf]

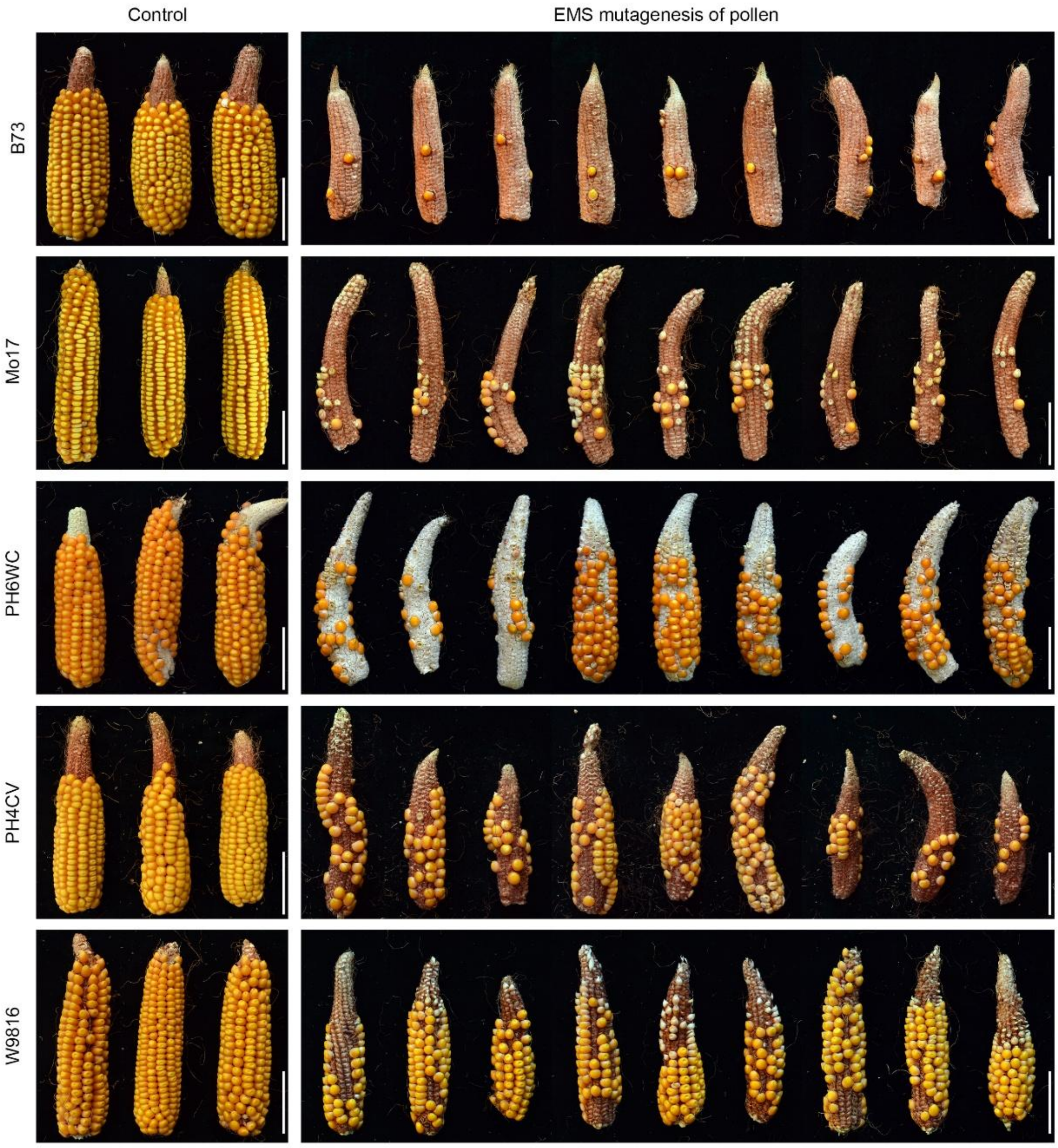

Figure S1. Harvested ears for different maize inbred lines after EMS mutagenesis of pollen

Five maize inbred lines were subjected to EMS mutagenesis of pollen. Left panel: self-pollinated ears without EMS mutagenesis (Control); right panel: harvested ears following EMS mutagenesis of maize pollen. Scale bar = 5 cm.

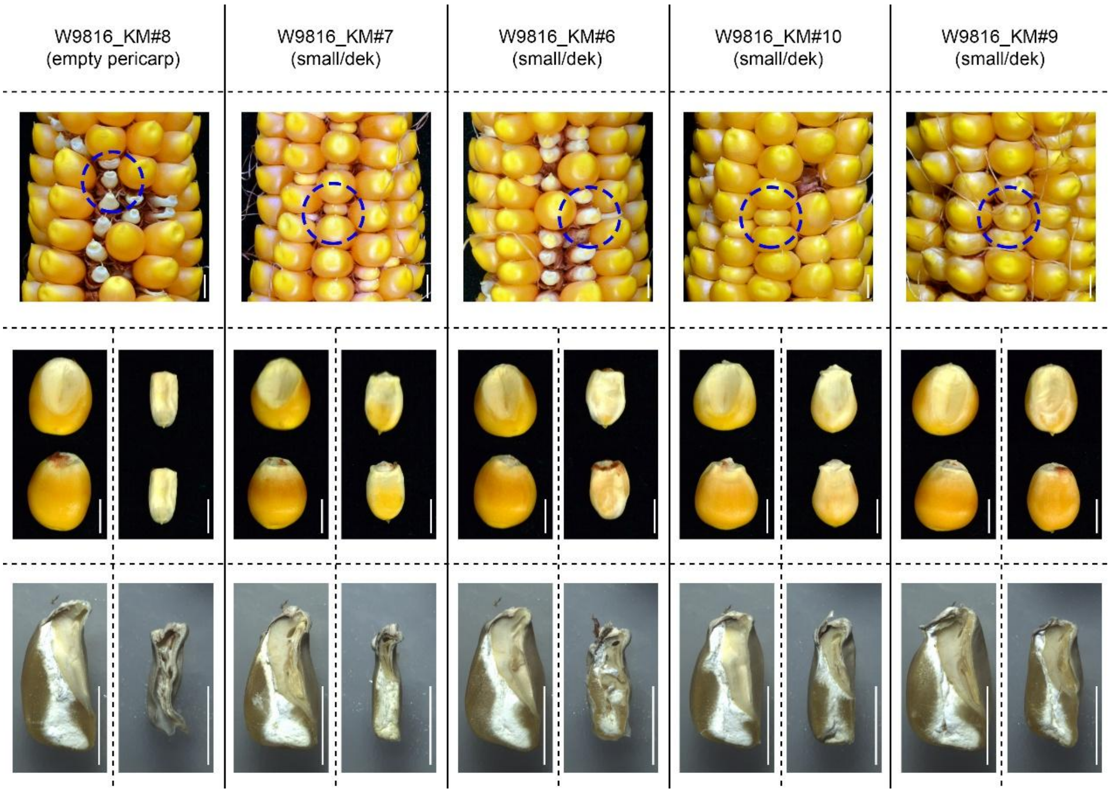

Figure S2. Representative kernel mutants in the W9816 background with varying defectiveness

For each mutant, the left panel shows a wild-type kernel, and the right panel shows a representative mutant kernel. Scale bar = 0.5 cm.

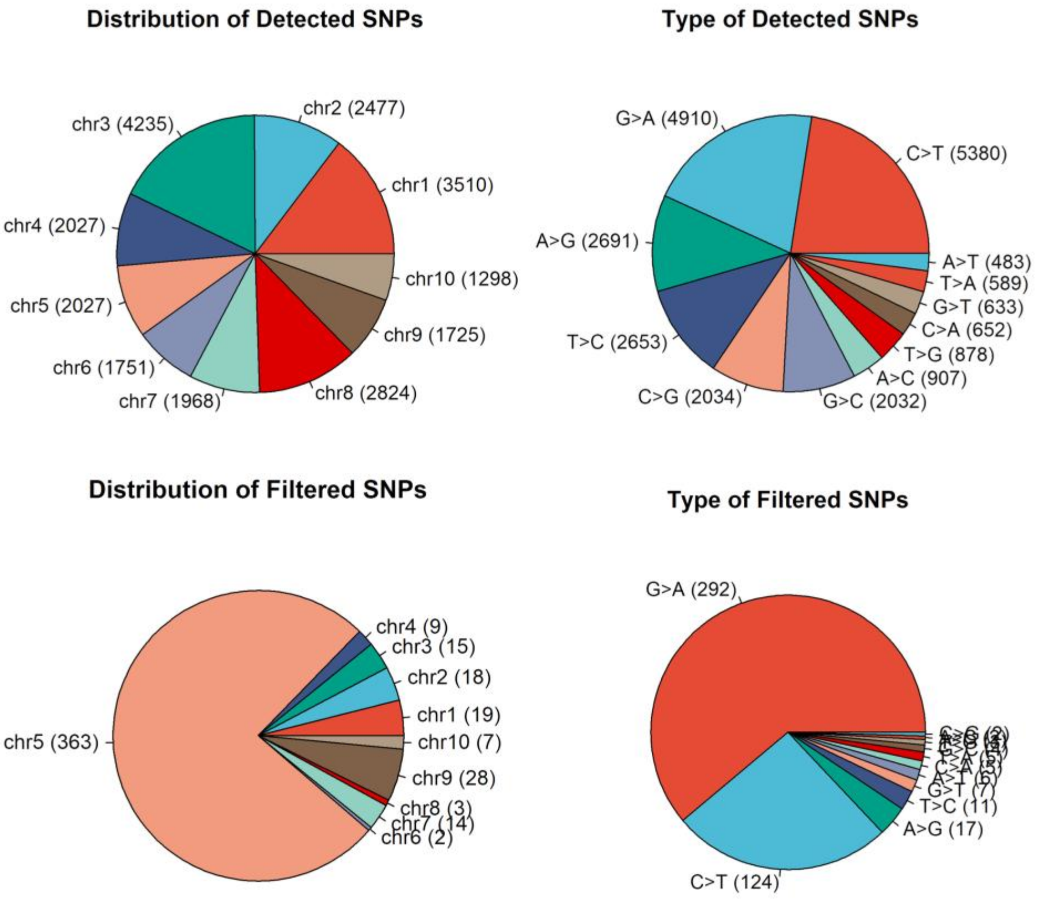

Figure S3. Distributions and types of detected and filtered SNPs for gene mapping of B73_KM#4

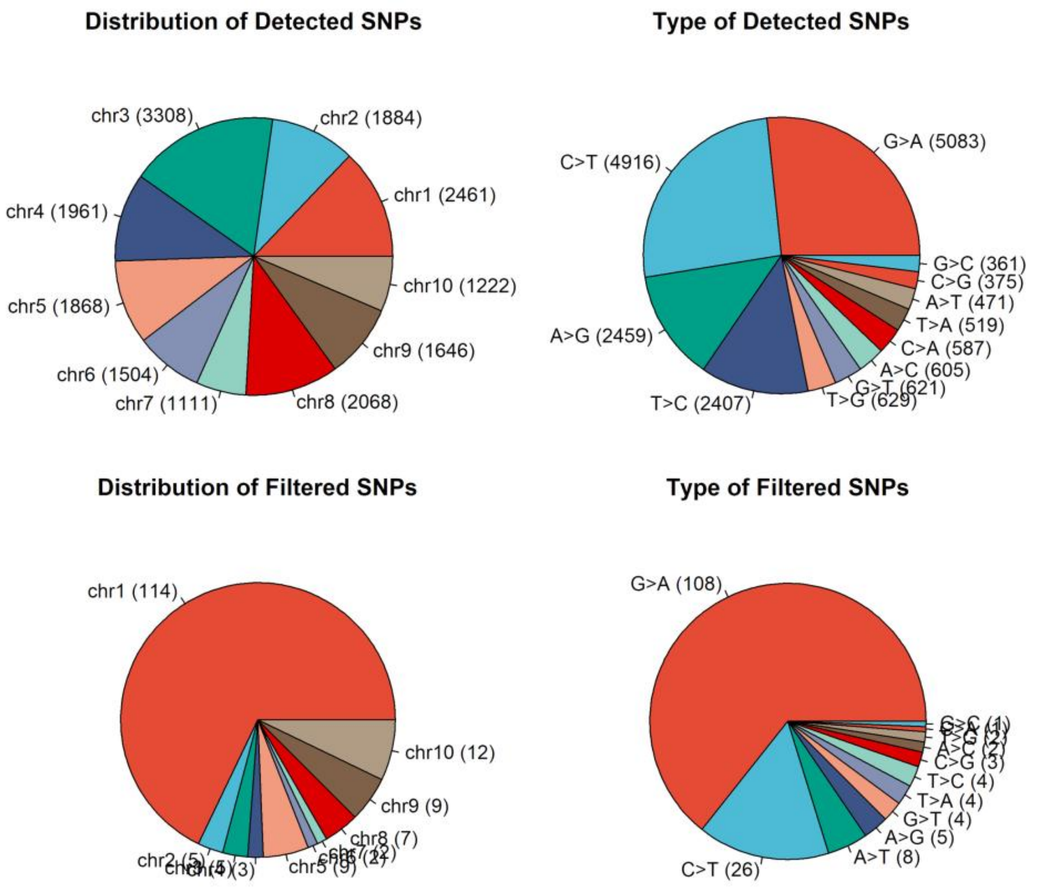

Figure S4. Distribution and types of detected and filtered SNPs for gene mapping of B73_KM#44

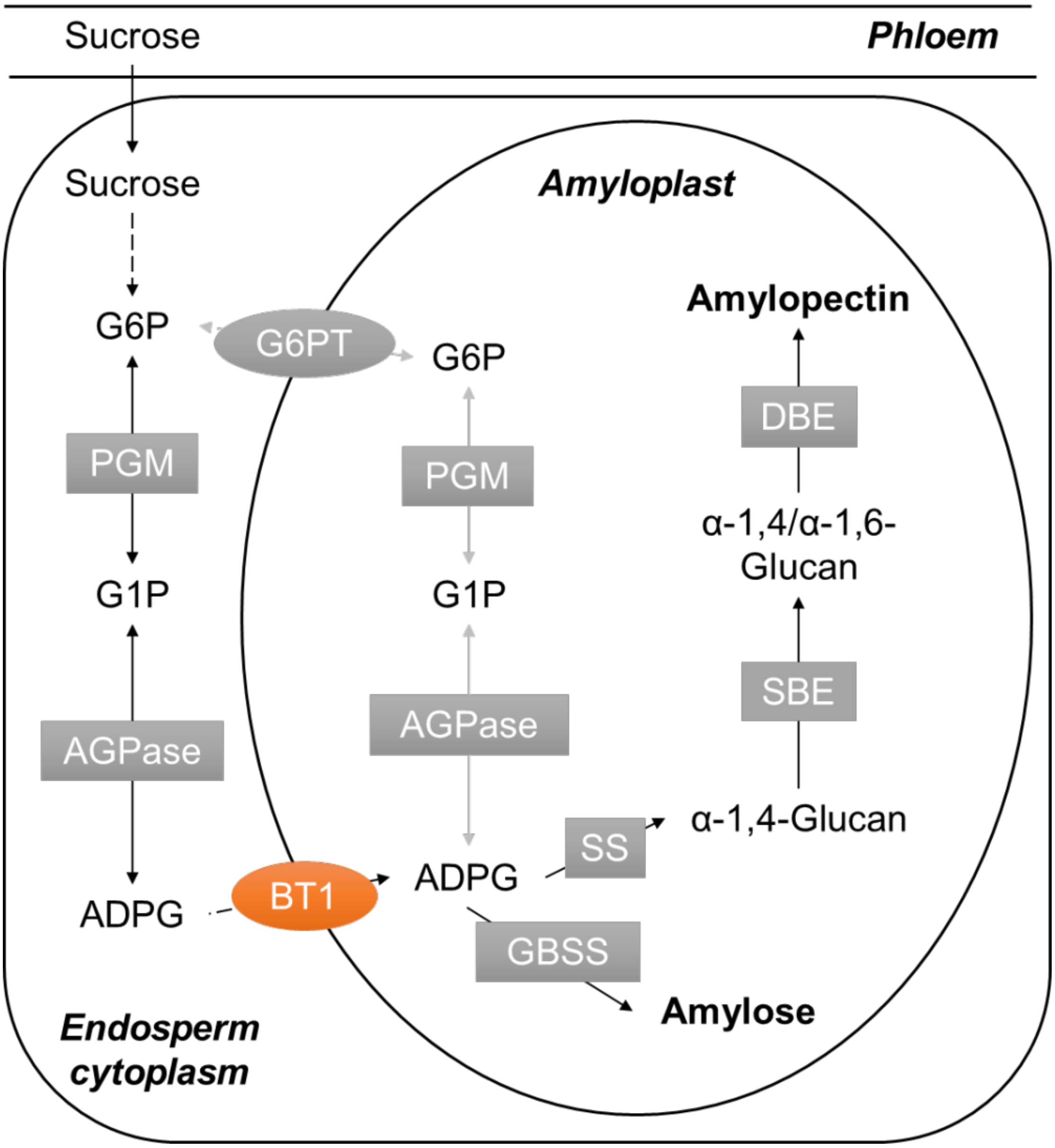

Figure S5. The involvement of ZmBT1 in maize endosperm starch biosynthesis

Sucrose is produced from the Calvin cycle in leaves and transported to the endosperm through the phloem. Abbreviations for involved components: G6P, glucose-6-phosphate; G1P, glucose-1-phosphate; ADPG, ADP-glucose. Involved proteins: PGM, phosphoglucomutase; AGPase, ADPG pyrophosphorylase; BT1, adenine nucleotide transporter; G6PT, G6P transporter; SS, starch synthase; SBE, starch branching enzyme; DBE, starch de-branching enzyme; GBSS, granule-bound starch synthase. The black arrows indicate the major pathway; the gray arrows indicate the mighty minor pathway.

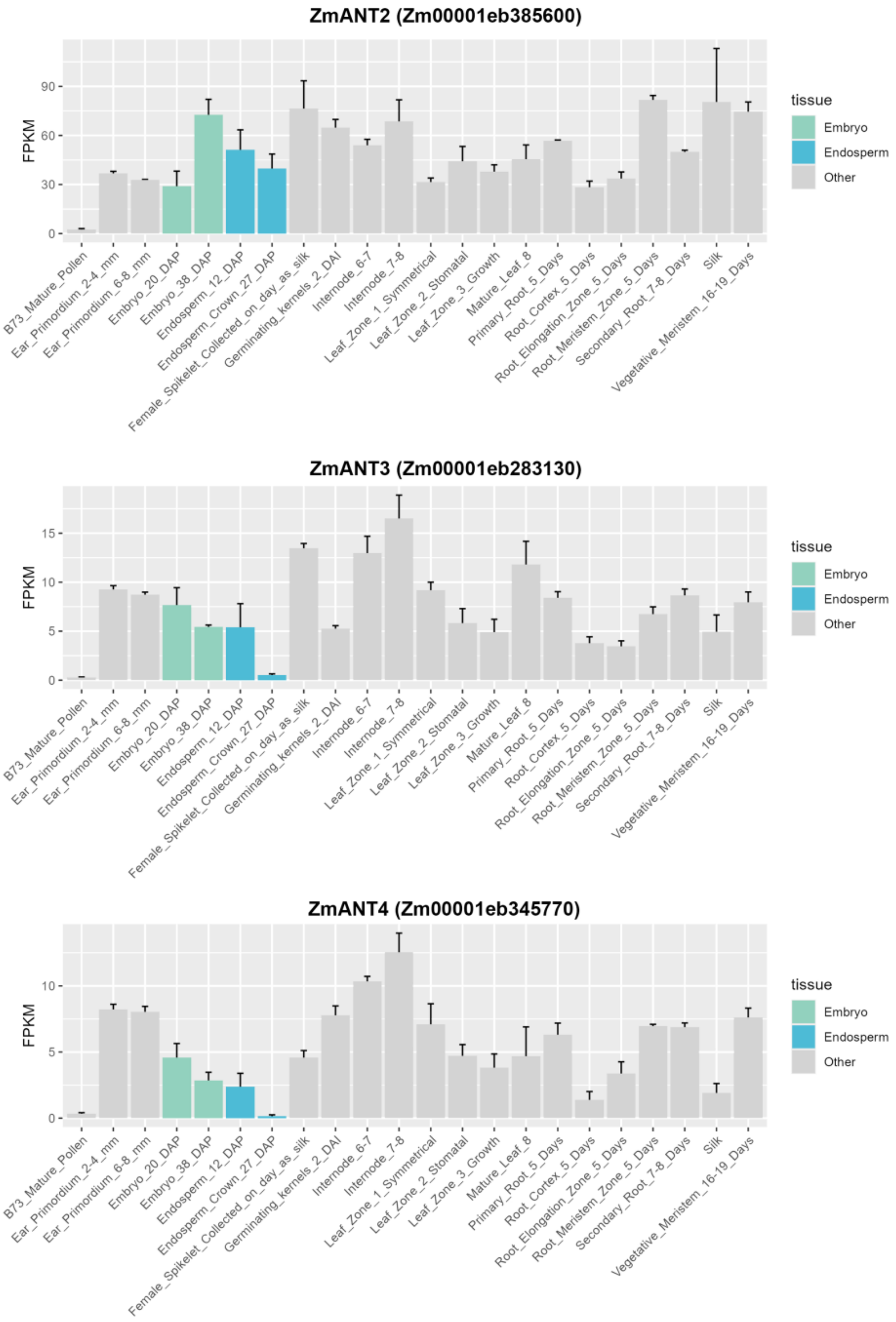

Figure S6. The spatial expression patterns of ZmANT2, ZmANT3, and ZmANT4

The RNA-Seq data spanning 22 maize tissues were obtained from qTeller-maizeGDB.
